# CDK4/6 inhibitor palbociclib overcomes acquired resistance to third‐generation EGFR inhibitor osimertinib in non‐small cell lung cancer (NSCLC)

**DOI:** 10.1111/1759-7714.13521

**Published:** 2020-07-16

**Authors:** Qiong Qin, Xiaoqing Li, Xingmei Liang, Lili Zeng, Jing Wang, Linlin Sun, Diansheng Zhong

**Affiliations:** ^1^ Department of Oncology Tianjin Medical University General Hospital Tianjin China; ^2^ Tianjin Lung Cancer Institute Tianjin Medical University General Hospital Tianjin China; ^3^ Tianjin Medical University Cancer Institute and Hospital Tianjin China

**Keywords:** Acquired resistance, CDK4/6 inhibitor palbociclib, cell cycle, third‐generation epidermal growth factor receptor (EGFR) tyrosine‐kinase inhibitor (TKI)

## Abstract

**Background:**

The third‐generation EGFR‐TKI, represented by osimertinib, has been widely used in clinical practice; however, resistance eventually emerges. At present, it remains unclear whether an abnormal cell cycle is involved in acquired resistance, and whether the combination of palbociclib (CDK4/6 inhibitor) and osimertinib can overcome the third‐generation TKI resistance.

**Methods:**

We established osimertinib‐resistant cells (H1975 OR) derived from *EGFR*‐mutant NSCLC cells H1975. Drug effects on cells were assessed with Cell Counting Kit‐8 (CCK8). Protein alterations were detected with western blot analysis. RT‐PCR was used to evaluate the differences of gene mRNA. Cell cycle distribution of H1975 S and H1975 OR cells was compared using flow cytometry.

**Results:**

Compared with H1975, the sensitivity of H1975OR to the CDK4/6 inhibitor was increased and the proportion of cells in G1 phase was decreased. The mRNA level of CDK4, CDK 6 and the protein level of CDK4, pRB were increased in H1975OR. In the H1975OR cells, palbociclib significantly increased the proportion of G1 phase cells. The combination of osimertinib and palbociclib synergistically decreased the survival of H1975OR by cell cycle arrest. Combined treatment was found to inhibit the initial phosphorylation of RB by inhibiting the function of CDK4/6, significantly reducing the level of p‐RB, and blocking cell proliferation.

**Conclusions:**

An osimertinib acquired resistance cell line (H1975 OR) was successfully established. The expression of cell cycle related genes was altered in H1975OR. The expression of CDK4 and the phosphorylation of Rb, the downstream molecule of CDK4/6, was increased in H1975OR cells. The combination of CDK4/6 inhibitor palbociclib and osimertinib could overcome the acquired resistance of osimertinib.

## Introduction

Lung cancer is still the most common malignant tumor and the leading cause of cancer‐related death worldwide and in China.^1^, ^2^ Non‐small cell lung cancer (NSCLC) accounts for about 92% of patients with lung cancer^3^ and in an Asian population 30%–52.4% of patients with NSCLC had *EGFR*‐activating mutations.^4^, ^5^, ^6^ Targeted treatment with tyrosine kinase inhibitors (TKIs) significantly prolonged the progression‐free survival (PFS) in patients with *EGFR* mutations,^7^, ^8^, ^9^ which was about 4.6–6.4 months in the era of chemotherapy to 9.8–13.1 months in first‐generation TKIs, and then to 18.9 months in the FLAURA study.

However, acquired resistance to EGFR TKIs is inevitable. After first‐generation EGFR TKI therapy, two studies reported that 50%–60% of patients were found to have T790M mutations,^10^, ^11^ and in another study the application of three generations of TKI osimertinib was still able to achieve more than 10 months of PFS in patients.^12^ However, acquired resistance to third‐generation EGFR TKI osimertinib eventually emerges, but the underlying mechanisms are not well understood.^11^, ^12^, ^13^, ^14^ The resistance mechanisms to osimertinib in C797X mutations has been reported to be only 10%–15%; other mechanisms include bypass activation, secondary mutation, tumor type conversion and so on.^13^, ^14^ In osimertinib acquired resistance, abnormal cell cycle regulation has been reported to be very common, including CCND1 amplification, CCND2 amplification, CCNE1, CDK4, CDK6 amplification and CDKN2A mutation.^13^, ^14^ About 10% of first‐line patients and 12% of second‐line patients treated with osimertinib were found to be resistant as a result of abnormal cell cycle regulation.^13^, ^14^ Therefore, abnormal cell cycle regulation is an important mechanism of acquired resistance to osimertinib.

Currently, CDK4/6 inhibitors are the main drugs approved by the FDA for cell cycle regulation,^15^, ^16^ which are palbociclib, rebociclib, and abemaciclib, and are mainly used in the treatment of HR positive breast cancer. Studies revealed that administration of a single CDK4/6 inhibitor combined with endocrine therapy achieved a good therapeutic effect, significantly prolonging the PFS and OS of patients.^17^, ^18^ CDK4/6 inhibitors have also been explored in the treatment of lung cancer, but the efficacy of single agent therapy has been found to be limited , with an ORR between 0%–8%, which is not high enough to instigate the wide use of a single CDK4/6 inhibitor in clinical practice.^19^, ^20^, ^21^

However, it is possible that CDK4/6 inhibitors may play a role in the treatment of TKI acquired resistant patients with *EGFR*‐sensitive mutations. A previous study revealed that gefitinib combined with CDK4/6 inhibitor palbociclib in gefitinib acquired resistance cells could partially reverse the resistance, which has been confirmed in PC9/AB2.^22^ It is not known whether the combination of osimertinib and palbociclib can overcome osimertinib acquired resistance. We established an osimertinib acquired resistance cell line H1975OR in our laboratory. The aim of this study was to use the H1975OR cell line to investigate whether the single agent palbociclib or its combination with osimeritinib could overcome acquired resistance and the potential mechanisms.

## Methods

### Cell lines and cell culture

Human lung adenocarcinoma cell lines H1975 (sensitive to osimertinib) and H1975OR (osimertinib resistant) were used in this study; all lines were preserved in our laboratory. Cells were cultured in RPMI‐1640 medium supplemented with 10% fetal bovine serum (Gibco, Grand Island, NY, USA) at 37°C in a humidified incubator with 5% CO_2_.

### Cell proliferation assay

The H1975 and H1975OR cells were seeded at a density of 1.0 × 10^4^ cells/well in a 96‐well plate for 48 and 72 hours. Cell Counting Kit‐8 (CCK‐8) solution (10 μL, B34302, Selleck Chemicals, Houston, TX, USA) was added to each well prior to the endpoint of incubation. The absorbance, which represented the cell count, was determined with a microculture plate reader at 450 nm.

### Flow cytometry analysis of cell cycle

To determine the effects of osimertinib and/or palbociclib on cell cycle, 1–2 × 10^5^ cells/well were seeded in six‐well plates and incubated for 12 hours. Cells were synchronized by starving them in serum‐free DMEM for 24 hours. Cells were then incubated with or without osimertinib and/or palbociclib for 24 hours, trypsinized, and fixed in 75% ice‐cold ethanol overnight. Cells were then treated with DNase‐free ribonuclease (Takara, Shiga, Japan), stained with 7AAD (Sigma‐Aldrich, MO, USA), and analyzed using ModFit LT software (Topsham, ME, USA).

### Reverse transcription and quantitative real‐time PCR

Total RNA was isolated from MSCs using Trizol (ThermoFisher Scientific, CA, USA) and an RNeasy Mini Kit (Qiagen), following the manufacturer's instructions. cDNA was synthesized using the M‐MLV Reverse Transcriptase Kit (Promega, WI, USA) according to the manufacturer's protocol. Quantitative real‐time PCR analysis was performed with ABI SYBR Green Master Mix (Thermofisher Scientific, CA, USA) in an ABI7500 real‐time PCR system according to the manufacturer's protocol. Each sample was run in triplicate for each gene. Transcript levels were normalized to the housekeeping gene phosphoglycerate kinase (PGK) and analyzed by the relative quantification 2‐ΔΔCt method. All gene primers were obtained from SBS (Beijing, China). We used Affymetrix GeneChip probe to test the gene changes in the RNA of H1975S and H1975OR cells. All the detected gene primers are shown in Table 1.

**Table 1:** The detected gene primers

Gene name	Forward prime (5′to3′)	Reverse prime (5′ to 3′)
CDK2	CCAGGAGTTACTTCTATGCCTGA	TTCATCCAGGGGAGGTACAAC
CDK4	TCAGCACAGTTCGTGAGGTG	GTCCATCAGCCGGACAACAT
CDK6	CCAGATGGCTCTAACCTCAGT	AACTTCCACGAAAAAGAGGCTT
CCND1	CAATGACCCCGCACGATTTC	CATGGAGGGCGGATTGGAA
CCNE	AAGGAGCGGGACACCATGA	ACGGTCACGTTTGCCTTCC
CDKN1a	CGATGGAACTTCGACTTTGTCA	GCACAAGGGTACAAGACAGTG
CDKN1b	TAATTGGGGCTCCGGCTAACT	TGCAGGTCGCTTCCTTATTCC
CDKN2a	GGGTTTTCGTGGTTCACATCC	CTAGACGCTGGCTCCTCAGTA
CDKN2b	GGAATGCGCGAGGAGAACAA	CATCATCATGACCTGGATCGC
CDKN2c	AAACTTGGAAATCCCGAGATTGC	CGAAACCAGTTCGGTCTTTCAA
CDKN2d	AGTCCAGTCCATGACGCAG	ATCAGGCACGTTGACATCAGC

### Western blotting

Protein samples were resolved by sodium dodecyl sulfate polyacrylamide gel electrophoresis and transferred to polyvinylidene fluoride membranes (Merck Millipore, Billerica, MA, USA). Membranes were blocked with fat‐free milk combined with tris‐buffered saline plus tween 20 for one hour at room temperature and then incubated with the appropriate primary antibody and horseradish peroxidase conjugated secondary antibodies. Imager was used to visualize the blots. The primary antibodies used in this study were as follows: anti‐CDK4 (#12790, 1:1000, cell signaling), Anti‐Cyclin D1 antibody (#2978, 1:1000, cell signaling), Anti‐phospho‐EGFR antibody (#47724, 1:1000, cell signaling), Anti‐Phospho‐RB (ser780, #9307, 1:1000, cell signaling), anti‐E2F1 (#666515,1:1000,Proteintech), CDKN2a (#80772, 1:1000, cell signaling); and β‐actin (ab92552, 1:1000).

### Statistical analysis

All data were analyzed by using GraphPad Prism 7.0 (GraphPad Software, La Jolla, CA). Independent‐samples *t*‐test were used to identify the statistical significance between groups. ANOVAs were used for analysis of more than two groups of data in all experiments. Significant differences between the means were considered. *P* < 0.05 was considered statistically significant.

## Results

### Generation and characterization of osimertinib acquired resistance H1975 cell line

**Figure 1:**
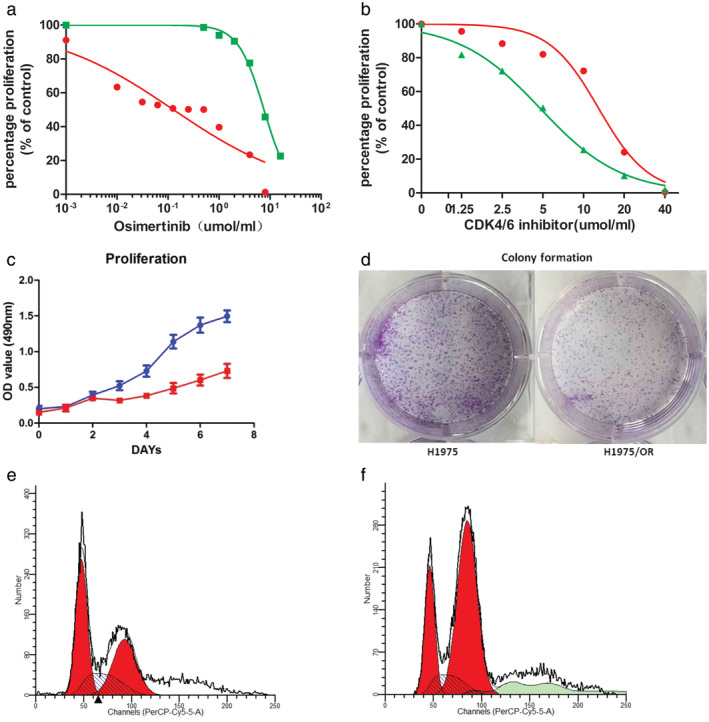
Establishment of osimertinib‐resistant H1975OR cells. (**a**) The IC50 of osimertinib in H1975S and H1975OR (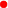) H1975s, and (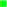) H1975OR; (**b**) The IC50 of palbociclib in H1975S and H1975OR (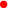) H1975S, and (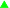) H975OR; (**c**, **d**). CCK8 and colony formation of H1975S (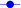) 1975, and (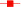) H1975 and H1975OR; (**e**) Cell cycle of H1975S (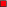) Dip G1, (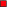) Dip G2, and (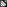) Dip S; (**f**) Cell cycle of H1975OR (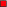) Dip G1, (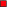) Dip G2, and (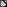) Dip S.

Acquired resistance of H1975S cells was induced by osimertinib. The drug concentration was gradually increased from 5 nM to 1.5 μM, and an H1975OR cell line was established about 22 weeks later.^23^ The changes in related genes in osimertinib acquired resistance, such as C797X, loss of T790M, amplification of MET, and *KRAS*, *BRAF*, *MEK* and *PI3K* mutations, were not found in H1975OR cells.^23^ The *EGFR L858R* and *T790M* mutation were still preserved in H1975OR cells.^23^ The inhibitory dose (IC50) of H1975OR cells was significantly higher than H1975S. The IC50 of the two groups of cell lines was 0.1276 and 7.593 µM  respectively (Fig 1(a)). The drug resistance index was 59.51, which was much higher than 10, indicating that the cell line was resistant.

Compared with H1975S, H1975OR was more sensitive to palbociclib, a CDK4/6 inhibitor. IC50 of palbociclib in H1975S cells was 12.94 μmol/mL, while which was 4.776 μmol/mL in H1975OR cells (Fig 1(b)). We used CCK8 assay and colony formation assay to evaluate the cell growth rate of H1975S and H1975OR. As shown in Fig 1(c), (d), H1975OR cells were slower to grow compared with H1975S.

In terms of cell cycle, compared with H1975S cell line, H1975OR cells line had fewer G1 phase and more G2 phase cells (Fig1(e),(f)). In the H1975S cells, G1 cell was 44.83%, while the H1975OR was 30.87%. Therefore, H1975OR cells were easier to through the R‐point block of G1 phase and to enter the S‐phase of cell synthesis than H1975S cells, which provided the possibility for cell cycle inhibitors.

### Combination of palbociclib increases the sensitivity to osimertinib in H1975‐OR cells

**Figure 2:**
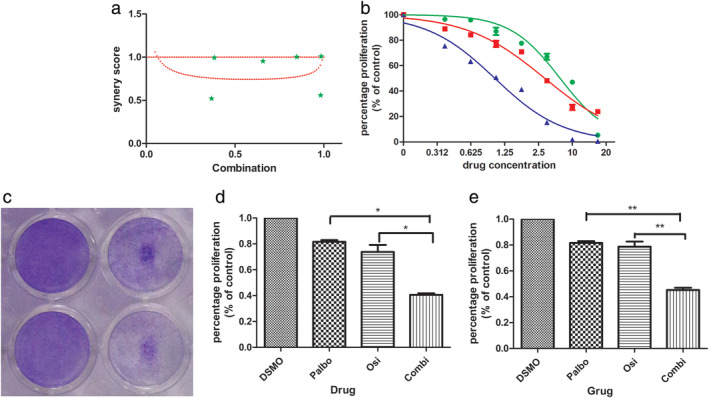
(**a**) Combined index (CI) of osimertinib and palbociclib (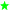) Measured value; (**b**) Inhibitory effect of osimertinib, palbociclib and combination in H1975OR cells (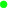) Osimertinib, (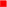) CDK4/6 inhibitor, and (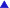) combination; (**c**) Crystal violet staining; (**d**, **e**) The bar graph shows the effect of osimertinib, palbociclib and combination on H1975OR proliferation ([**c**] Osi 2.5 μM, Palbociclib 1.25 μM; Combi 2:1, *P* < 0.05; [**d**] Osi 1.25 µM, Palbociclib 1.25 µM; Combi 1:1, *P* < 0.05).

Next, we sought to investigate whether the combination of palbociclib could increase the sensitivity to osimertinib in H1975OR cells. We found that the combination of palbociclib significantly increased the inhibitory effect of osimertinib in H1975OR (Fig 2(b)), with a combined index (CI) ≤1 (Fig 2(a)), indicating a synergistic effect of the two drugs. The efficacy of combining palbociclib with osimertinib in suppressing cell growth was further validated by crystal violet staining (Fig 2(c)). It could obviously inhibit the proliferation of H1975OR when the drug concentration of osimertinib and palbociclib were close drug concentration in human plasma. (According to the recommended dosage of 80 mg QD of osimertinib, the drug concentration in human plasma is about 0.8 µM, which is close to the effective drug concentration of the combination). Specifically, the survival rate of osimertinib (2.5 μM) was 73.80% (SD + 5.41%), the survival rate of palbociclib (1.25 μM) was 81.67% (SD + 1.29%), the survival rate of a combination was 40.67% (SD + 1.13%); the survival rate of osimertinib (1.25 μM) was 78.67% (SD + 3.97%), the survival rate of a combination was 45.2% (SD): difference + 1.79%) (Fig 2(c),(d), *P* < 0.05). Together, these results indicate that the combination of palbociclib could increase the sensitivity to osimertinib in H1975OR cells.

### Effect of palbociclib on cell cycle of H1975S and H1975OR were different

**Figure 3:**
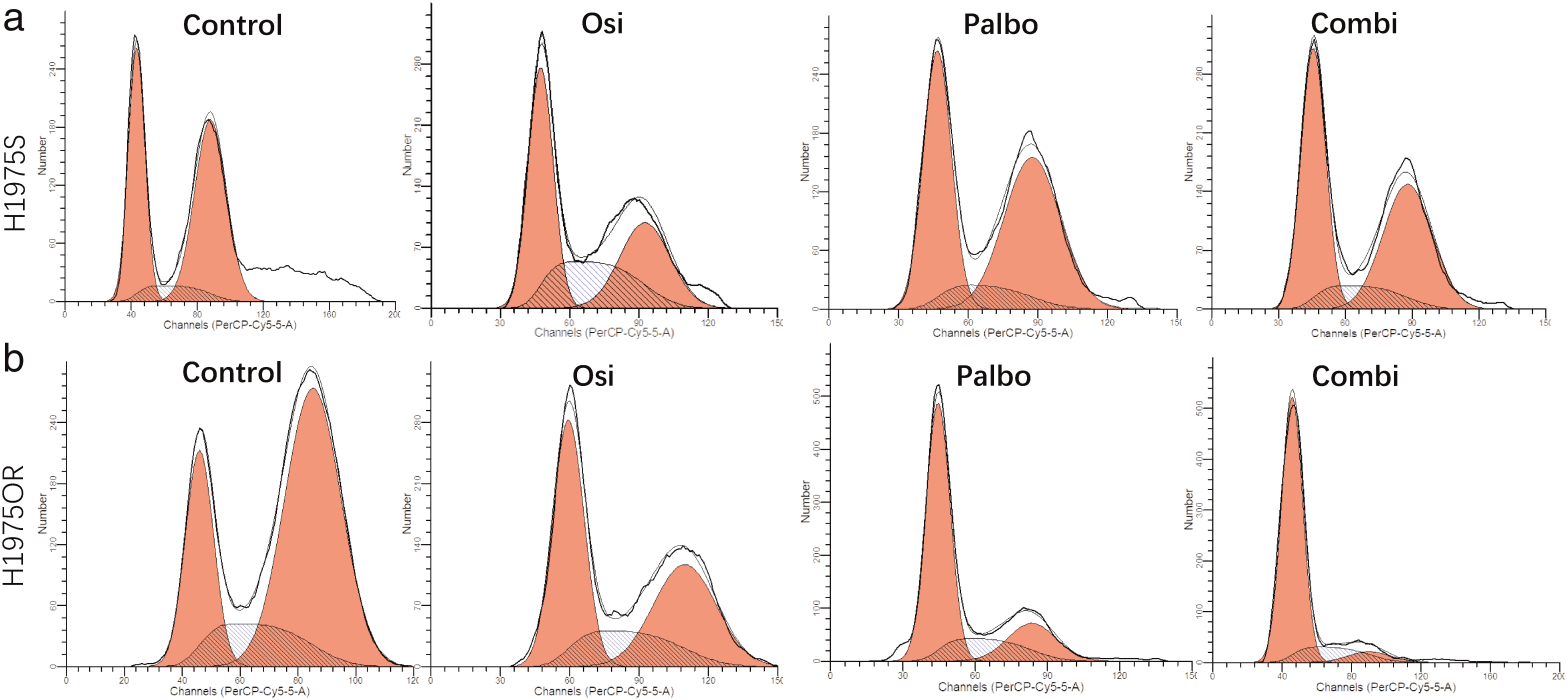
Cell cycle changes of (**a**) H1975S and (**b**) H1975OR (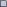) Debris, (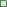) Aggregates, (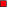) Dip G1, (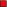) Dip G2, and (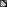) Dip S; (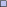) Debris, (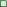) Aggregates, (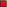) Dip G1, (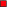) Dip G2, and (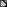) Dip S; (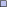) Debris, (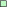) Aggregates, (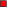) Dip G1, (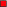) Dip G2, and (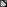) Dip S; and (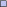) Debris, (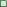) Aggregates, (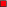) Dip G1, (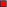) Dip G2, and (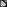) Dip S cells with control, osimertinib, palbociclib and combination (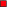) Dip G1, (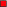) Dip G2, and (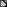) Dip S; (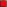) Dip G1, (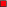) Dip G2, and (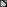) Dip S; (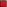) Dip G1, (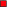) Dip G2, and (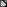) Dip S; and (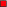) Dip G1, (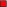) Dip G2, and (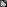) Dip S.

There were differences between H1975S and H1975OR in the use of palbociclib and in the combination of palboclib and osimertinib. The effect of palbociclib on cell cycle of H1975S cells was not obvious. In H1975S cells, the proportion was 37.24% in control, 37.25% osimertinib, 41.91% in palbociclib, 43.71% combination (*P* > 0.05). However, in H1975OR cells, the proportion was 28.32% in control, 42.47% osimertinib, 64.86% in palbociclib, 76.49% in combination (Fig 3).

### H1975S expression of cell cycle related genes were altered in H1975‐OR cells

**Figure 4:**
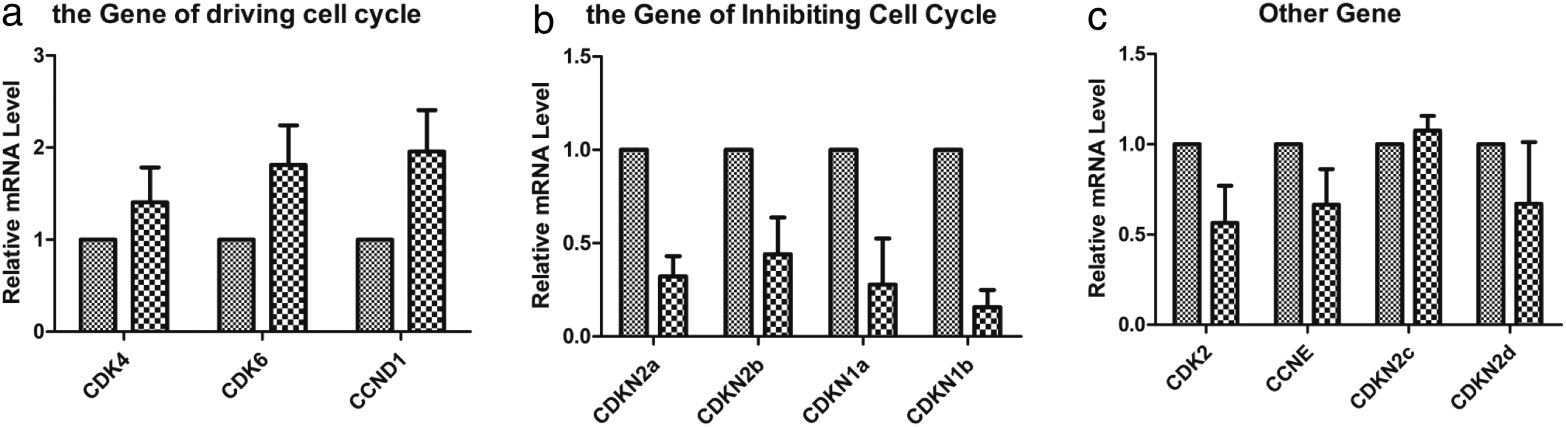
The differences of mRNA between H1975S and H1975OR. (**a**) mRNA levels of genes related to cell cycle promotion (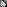) H1975S, and (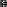) H1975OR; (**b**) mRNA levels of genes related to cell cycle inhibition (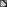) H1975S, and (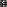) H1975OR; (**c**) mRNA levels of other cell cycle related genes (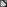) H1975S, and (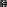) H1975OR.

To reveal the underlying molecular mechanism, we tested whether there were differences in the level of cell cycle related genes between H1975S and H1975OR cells. The genes included CDK2, CDK4, CDK6, CCND1, CCNE, CDKN1a, CDKN1b, CDKN2a, CDKN2b, CDKN2c, CDKN2d, and the internal reference gene was GADPH. We found that the mRNA levels of CDK4, CDK6 and CCND1 driving cell cycle were upregulated (*P* = 0.055, 0.042, 0.039), while the mRNA levels of CDKN2A, CDKN2b, CDKN1a, CDKN1b (*P* = 0.038, 0.044,0.036 and 0.023) inhibiting the cell cycle were significantly downregulated. There were no significant changes in other genes such as CDK2, CCNE, CDKN2c, and CDKN2d (Fig 4).

### Expression of CDK4, E2F1 and phosphorylation of RB increased in H1975‐OR cells

**Figure 5:**
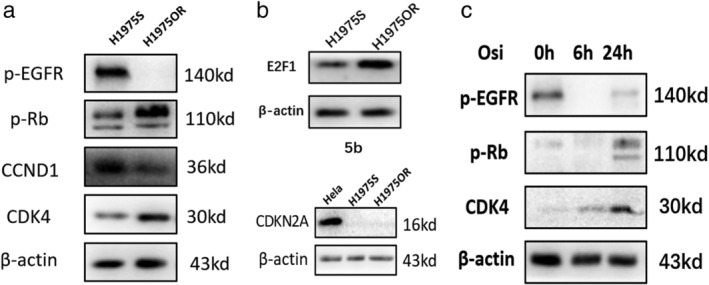
(**a**, **b**, **c**) Western blot analysis for detection of p‐EGFR (Y‐1068), p‐RB (S‐780), CDK4, CCND1, E2F1, and CDKN2A; (**d**) H1975S cells treated with osimertinib for 6 and 24 hours.

Compared with H1975S cells, the phosphorylation of RB (p‐RB) was higher in H1975OR cells. Further analysis of cell cycle‐related proteins CDK4, cyclin D1 (CCND1) and CDKN2A showed that the expression of CDK4 was significantly increased in H1975OR cells (Fig 5(a)). At the same time, E2F1 was higher in H1975OR compared with H1975S (Fig 5(b)). The level of p‐EGFR (Y‐1068) in H1975OR was significantly lower than that in H1975S, suggesting that EGFR activity is inhibited in H1975OR. However, CDKN2A, an endogenous inhibitor that inhibited the cell cycle process, was not found expressed in both sensitive and resistant cell lines with HeLa cells as a positive control (Fig 5(c)) In sensitive cell lines, CDK4 expression and the phosphorylation of RB (p‐RB) (S‐780) in H1975S cells were increased upon treatment with osimertinib in a time‐dependent manner (Fig 5(d)). Thus, our results suggest that the upregulation of CDK4 and hyperphophorylation of Rb might contribute to osimertinib resistance in H1975OR.

### Combination of osimertinib and palbociclib reduces p‐RB and increases cell block in G1 phase

**Figure 6:**
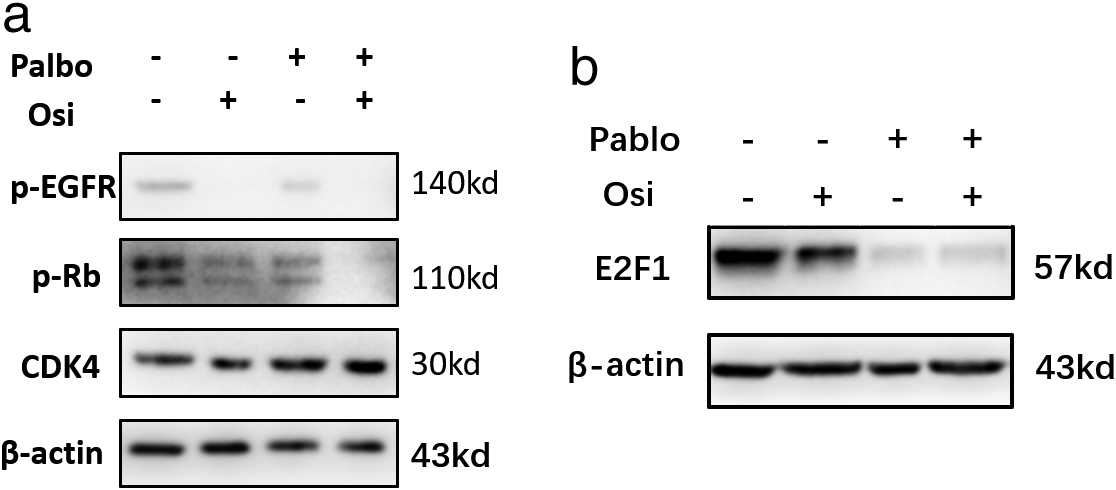
Changes of related proteins in H1975OR cells treated with osimertinib, palbociclib and combination.

Administration of single doses of palbociclib or osimertinib could downregulate the level of p‐RB, E2F1, but the p‐RB and E2F1 decreased significantly after the combined treatment (Fig 6(a),(b)). There was little change in the CDK4 protein of H1975OR cells treated with osimertinib. However, the CDK4 protein treated with palbociclib increased slightly, while feedback expression increased after the combination of osimertinib and palbociclib. The expression of p‐RB decreased after treatment with osimertinib or palbociclib. G1 phase arrest of the cells increased significantly, which was consistent with the cell cycle analysis.

## Discussion

A series of studies have been published on acquired resistance to osimertinib.^24^, ^25^, ^26^, ^27^, ^28^, ^29^, ^30^ The L858R mutation combined with T790M mutation was found to be more similar in real‐world patients who acquired T790M mutation after first‐ or second‐generation TKIs.^10^, ^11^ In this study, common osimertinib resistance mechanisms, such as C797X, loss of T790M, amplification of MET, and the mutation of *KRAS*, *BRAF*, *MEK* and *PI3K*, were not found in H1975OR cells,^23^, ^30^ which is similar to previous studies^13^ and represents a more common clinical situation. We found that the sensitivity of H1975OR cells to CDK4/6 inhibitor palbociclib increased, and combination of palbociclib and osimertinib could overcome drug resistance.

In this study, the IC50 of osimertinib was 7.593 μM, which was higher than that of the H1975OR osimertinib‐resistant cells (0.8–2.5 μM) established by other laboratories,^29^ which might be related to our H1975OR‐resistant cell line after a longer time of drug resistance screening. The establishment of our drug‐resistant cell line took about 22 weeks, while other studies report that it took about 6–12 weeks.^25^, ^29^

Shah *et al*. found that for H1975, osimertinib and rociletinib were used to induce drug resistance, respectively, and secondary TPX2 overactivation caused the increase of AURKA phosphorylation and BIM phosphorylation, which led to BIM ubiquitin degradation unable to play its role in inducing apoptosis.^25^, ^29^ AURKA inhibitor MLN8237 could block the phosphorylation of BIM, and BIM could induce apoptosis again to reverse the drug resistance of TKI of the third‐generation osimertinib. Aurora kinase inhibitors were found to suppress this adaptive survival program, thereby increasing the magnitude and duration of EGFR inhibitor response in preclinical models.^29^ Hayakawa *et al*. showed that the activation of IGF1R was involved in the acquired resistance of osimertinib.^25^ Both the knockdown IGF1R and IGF1R inhibitor could reverse the drug resistance and reconstruct the sensitivity of osimertinib.^24^, ^25^ In this study, there was no detection of AURKA kinase and IGF1R, so it is impossible to determine whether there was overactivation of AURKA and IGF1R.

Other possible mechanisms, including AXL and HER3 activation, have been found in the study of drug‐resistant cell lines induced by osimertinib. AXL inhibitor has been reported to be used in vitro and in vivo to delay the development of drug resistance and included including reverse acquired resistance.^26^, ^31^ It was also considered that ERK or MEK inhibitors combined with osimertinib could overcome osimertinib resistance, mainly through the regulation of BIM mediated apoptosis.^27^, ^32^ Marcar *et al*. found that the activity of RAC1 in the TKI‐resistant cell line was enhanced by PARP‐1, which restricted its production of ROS mediated by NOX, and then weakened the role of ROS killing cells;^28^ PARP inhibitors could enhance ROS production and reverse drug resistance by inhibiting PARP‐1 activity.^28^ However, these studies did not include the resistant cell lines produced by H1975S. There was no clear evidence of those mechanisms in previous studies such as FLAURA and AURA3,^13^, ^14^ and it was not clear whether it was the real mechanism of acquired resistance of osimertinib.

According to FLAURA, AURA3 and real‐world studies, about 10%–12% of the patients had overexpression of CDK4, CDK6, and/or CCND1, CCND2 and low expression, or deletion of CDKN2a.^13^, ^14^, ^33^ Therefore, abnormal cell cycle regulation is very common in osimertinib resistance. The presence of these factors was also a potential benefit basis for CDK4/6 inhibitors palbociclib.^16^, ^34^, ^35^ In this study, it was found that the upregulation of CDK4 and the deficiency of CDKN2a in H1975OR cells, provided a theoretical basis for using palbociclib. In our study, we confirmed that palbociclib inhibited CDK4/6 to activate CCND1, and then inhibited RB phosphorylation, blocked the cell cycle significantly in the G1 phase, and controlled cell proliferation. The combination of palbociclib and osimertinib resulted in G1 phase arrest which had a better effect on the proliferation than any single drug in H1975OR. In previous studies, the first‐generation TKI gefitinib was used to induce pC9 cell line to produce the drug‐resistant cell line pC9/AB2, and the combination of CDK4/6 inhibitor and gefitinib could reverse the resistance.^22^ In the study, the IC50 of gefitinib was 211.2 μM, CDK4/6 inhibitor palbociclib IC50 33.35 μM, combination of gefitinib 16 μM and palbociclib 8 μM PC9/AB2 cell line. However, these drug concentrations were significantly higher than that in human blood, so it might limit further clinical exploration. In our study, the IC50 of osimertinib was 7.593 μM, palbociclib 4.766 μM, the combination treatment was 0.625–1.25 μM and palbociclib 1.25 μmol/L, which is close to concentration in human blood.^12^, ^21^ This will provide a better prospect for the clinical application of the drug combination.

At the same time, there are many deficiencies in our study. First, the combination of palbociclib and osimertinib was only explored in H1975OR cells. It is difficult to confirm whether this combination therapy could overcome the acquired resistance by osimertinib in other cell lines, and it is therefore necessary to further expand studies to include other cell lines such as PC9 and HCC827. Second, our study was only verified in cell lines, but no further animal experiments were carried out, so further exploration is needed to verify the efficacy in animal experiments. Third, the study was not carried out in human subjects, and the human environment is complex, including essential differences with cell lines in drug metabolism, tumor microenvironment, etc. Therefore, further exploration is needed.

In conclusion, in this study it was confirmed that the sensitivity of H1975OR to CDK4/6 inhibitor palbociclib was increased. The combination of CDK4/6 inhibitor palbociclib and osimertinib downregulated the phosphorylation of RB, blocked the acquired resistant cells in G1 phase, and controlled cell proliferation.

Here, we showed that expression of CDK4 and phosphorylation of Rb were increased in osimertinib‐resistant lung cancer cells. Inhibition of CDK4/6 with palbociclib significantly enhanced the sensitivity of the resistant cells to osimertinib. Our study sheds new light onto the mechanisms of osimertinib resistance and provides a rationale for targeting CDK4/6 as a potential therapy in overcoming third‐generation EGFR‐TKIs in lung cancer treatment.

## Disclosure

The authors declare that there are no conflicts of interest.

## References

[1] Chen W , Zheng R , Baade PD *et al* Cancer statistics in China, 2015. CA Cancer J Clin 2016; 66 (2): 115–32. 26808342 10.3322/caac.21338

[2] Bray F , Ferlay J , Soerjomataram I , Siegel RL , Torre LA , Jemal A . Global cancer statistics 2018: GLOBOCAN estimates of incidence and mortality worldwide for 36 cancers in 185 countries. CA Cancer J Clin 2018; 68 (6): 394–424. 30207593 10.3322/caac.21492

[3] Detterbeck FC , Franklin WA , Nicholson AG *et al* The IASLC lung cancer staging project: Background data and proposed criteria to distinguish separate primary lung cancers from metastatic foci in patients with two lung tumors in the forthcoming eighth edition of the TNM classification for lung cancer. J Thorac Oncol 2016; 11 (5): 651–65. 26944304 10.1016/j.jtho.2016.01.025

[4] Zhou C , Wu YL , Chen G *et al* Erlotinib versus chemotherapy as first‐line treatment for patients with advanced EGFR mutation‐positive non‐small‐cell lung cancer (OPTIMAL, CTONG‐0802): A multicentre, open‐label, randomised, phase 3 study. Lancet Oncol 2011; 12 (8): 735–42. 21783417 10.1016/S1470-2045(11)70184-X

[5] Tu HY , Ke EE , Yang JJ *et al* A comprehensive review of uncommon EGFR mutations in patients with non‐small cell lung cancer. Lung Cancer 2017; 114: 96–102. 29173773 10.1016/j.lungcan.2017.11.005

[6] Pi C , Xu CR , Zhang MF *et al* EGFR mutations in early‐stage and advanced‐stage lung adenocarcinoma: Analysis based on large‐scale data from China. Thorac Cancer 2018; 9 (7): 814–9. 29722148 10.1111/1759-7714.12651 PMC6026603

[7] Mok TS , Wu YL , Thongprasert S *et al* Gefitinib or carboplatin‐paclitaxel in pulmonary adenocarcinoma. N Engl J Med 2009; 361 (10): 947–57. 19692680 10.1056/NEJMoa0810699

[8] Fukuoka M , Wu YL , Thongprasert S *et al* Biomarker analyses and final overall survival results from a phase III, randomized, open‐label, first‐line study of gefitinib versus carboplatin/paclitaxel in clinically selected patients with advanced non‐small‐cell lung cancer in Asia (IPASS). J Clin Oncol 2011; 29 (21): 2866–74. 21670455 10.1200/JCO.2010.33.4235

[9] Ramalingam SS , Vansteenkiste J , Planchard D *et al* Overall survival with osimertinib in untreated, EGFR‐mutated advanced NSCLC. N Engl J Med 2020; 382 (1): 41–50. 31751012 10.1056/NEJMoa1913662

[10] Kobayashi S , Boggon TJ , Dayaram T *et al* EGFR mutation and resistance of non‐small‐cell lung cancer to gefitinib. N Engl J Med 2005; 352 (8): 786–92. 15728811 10.1056/NEJMoa044238

[11] Yu HA , Arcila ME , Rekhtman N *et al* Analysis of tumor specimens at the time of acquired resistance to EGFR‐TKI therapy in 155 patients with EGFR‐mutant lung cancers. Clin Cancer Res 2013; 19 (8): 2240–7. 23470965 10.1158/1078-0432.CCR-12-2246 PMC3630270

[12] Mok TS , Wu Y , Ahn M *et al* Osimertinib or platinum‐pemetrexed in EGFR T790M‐positive lung Cancer. N Engl J Med 2017; 376 (7): 629–40. 27959700 10.1056/NEJMoa1612674 PMC6762027

[13] Leonetti A , Sharma S , Minari R , Perego P , Giovannetti E , Tiseo M . Resistance mechanisms to osimertinib in EGFR‐mutated non‐small cell lung cancer. Br J Cancer 2019; 121 (9): 725–37. 31564718 10.1038/s41416-019-0573-8 PMC6889286

[14] Blakely CM , Watkins T , Wu W *et al* Evolution and clinical impact of co‐occurring genetic alterations in advanced‐stage EGFR‐mutant lung cancers. Nat Genet 2017; 49 (12): 1693–704. 29106415 10.1038/ng.3990 PMC5709185

[15] Klein ME , Kovatcheva M , Davis LE , Tap WD , Koff A . CDK4/6 inhibitors: The mechanism of action may not be as simple as once thought. Cancer Cell 2018; 34 (1): 9–20. 29731395 10.1016/j.ccell.2018.03.023 PMC6039233

[16] Sherr CJ , Beach D , Shapiro GI . Targeting CDK4 and CDK6: From discovery to therapy. Cancer Discov 2016; 6 (4): 353–67. 26658964 10.1158/2159-8290.CD-15-0894 PMC4821753

[17] Finn RS , Martin M , Rugo HS *et al* Palbociclib and letrozole in advanced breast cancer. N Engl J Med 2016; 375 (20): 1925–36. 27959613 10.1056/NEJMoa1607303

[18] Goetz MP , Toi M , Campone M *et al* MONARCH 3: Abemaciclib as initial therapy for advanced breast cancer. J Clin Oncol 2017; 35 (32): 3638–46. 28968163 10.1200/JCO.2017.75.6155

[19] Patnaik A , Rosen LS , Tolaney SM *et al* Efficacy and safety of Abemaciclib, an inhibitor of CDK4 and CDK6, for patients with breast cancer, non‐small cell lung cancer, and other solid tumors. Cancer Discov 2016; 6 (7): 740–53. 27217383 10.1158/2159-8290.CD-16-0095

[20] Puyol M , Martin A , Dubus P *et al* A synthetic lethal interaction between K‐Ras oncogenes and Cdk4 unveils a therapeutic strategy for non‐small cell lung carcinoma. Cancer Cell 2010; 18 (1): 63–73. 20609353 10.1016/j.ccr.2010.05.025

[21] Flaherty KT , Lorusso PM , Demichele A *et al* Phase I, dose‐escalation trial of the oral cyclin‐dependent kinase 4/6 inhibitor PD 0332991, administered using a 21‐day schedule in patients with advanced cancer. Clin Cancer Res 2012; 18 (2): 568–76. 22090362 10.1158/1078-0432.CCR-11-0509

[22] Liu M , Xu S , Wang Y *et al* PD 0332991, a selective cyclin D kinase 4/6 inhibitor, sensitizes lung cancer cells to treatment with epidermal growth factor receptor tyrosine kinase inhibitors. Oncotarget 2016; 7 (51): 84951–64. 27825114 10.18632/oncotarget.13069 PMC5356711

[23] Li X. Establishment of third generation EGFR‐TKI Osimertinib acquired resistant NSCLC cell line and preliminary study on resistance mechanism (Doctor thesis). Tianjin Medical University, 2019.

[24] Manabe T , Yasuda H , Terai H. *et al* IGF2 autocrine‐mediated IGF1R activation is a clinically relevant mechanism of osimertinib resistance in lung cancer. Mol Cancer Res 2020; **18**: 549–559. 10.1158/1541-7786.MCR-19-0956 31941753

[25] Hayakawa D , Takahashi F , Mitsuishi Y *et al* Activation of insulin‐like growth factor‐1 receptor confers acquired resistance to osimertinib in non‐small cell lung cancer with EGFR T790M mutation. Thorac Cancer 2020; 11 (1): 140–9. 31758670 10.1111/1759-7714.13255 PMC6938756

[26] Taniguchi H , Yamada T , Wang R *et al* AXL confers intrinsic resistance to osimertinib and advances the emergence of tolerant cells. Nat Commun 2019; 10 (1): 259. 30651547 10.1038/s41467-018-08074-0 PMC6335418

[27] Li Y , Zang H , Qian G , Owonikoko TK , Ramalingam SR , Sun SY . ERK inhibition effectively overcomes acquired resistance of epidermal growth factor receptor‐mutant non‐small‐cell lung cancer cells to osimertinib. Cancer 2020; 126(6): 1339–50. 31821539 10.1002/cncr.32655 PMC7050394

[28] Marcar L , Bardhan K , Gheorghiu L *et al* Acquired resistance of EGFR‐mutated lung Cancer to tyrosine kinase inhibitor treatment promotes PARP inhibitor sensitivity. Cell Rep 2019; 27 (12): 3422–32. 31216465 10.1016/j.celrep.2019.05.058 PMC6624074

[29] Shah KN , Bhatt R , Rotow J *et al* Aurora kinase A drives the evolution of resistance to third‐generation EGFR inhibitors in lung cancer. Nat Med 2019; 25 (1): 111–8. 30478424 10.1038/s41591-018-0264-7 PMC6324945

[30] Husain H , Scur M , Murtuza A , Bui N , Woodward B , Kurzrock R . Strategies to overcome bypass mechanisms mediating clinical resistance to EGFR tyrosine kinase inhibition in lung Cancer. Mol Cancer Ther 2017; 16 (2): 265–72. 28159915 10.1158/1535-7163.MCT-16-0105

[31] Zhang Z , Lee JC , Lin L *et al* Activation of the AXL kinase causes resistance to EGFR‐targeted therapy in lung cancer. Nat Genet 2012; 44 (8): 852–60. 22751098 10.1038/ng.2330 PMC3408577

[32] Tao Z , Le Blanc JM , Wang C *et al* Coadministration of trametinib and palbociclib radiosensitizes KRAS‐mutant non‐small cell lung cancers in vitro and in vivo. Clin Cancer Res 2016; 22 (1): 122–33. 26728409 10.1158/1078-0432.CCR-15-0589

[33] Papadimitrakopoulou VA , Han JY , Ahn MJ *et al* Epidermal growth factor receptor mutation analysis in tissue and plasma from the AURA3 trial: Osimertinib versus platinum‐pemetrexed for T790M mutation‐positive advanced non‐small cell lung cancer. Cancer‐Am Cancer Soc 2020; 126 (2): 373–80. 10.1002/cncr.32503 31769875

[34] Li P , Zhang X , Gu L , Zhou J , Deng D . P16 methylation increases the sensitivity of cancer cells to the CDK4/6 inhibitor palbociclib. PLOS One 2019; 14 (10): e223084. 10.1371/journal.pone.0223084 PMC6814222 31652270

[35] Gopalan PK , Villegas AG , Cao C *et al* CDK4/6 inhibition stabilizes disease in patients with p16‐null non‐small cell lung cancer and is synergistic with mTOR inhibition. Oncotarget 2018; 9 (100): 37352–66. 30647837 10.18632/oncotarget.26424 PMC6324768

